# Medicine, Pathological, Practical, and Therapeutical

**Published:** 1836-04

**Authors:** 


### MEDICINE,

### PATHOLOGICAL, PRACTICAL, AND THERAPEUTICAL.

*On Diseases of the Spleen, as they occur in India.* By J. O. VOIGT, Physician to the Danish Establishment of Frederiksnagor (Serampore).

CHRONIC diseases of the spleen are so frequent and fatal throughout India, especially in the low marshy provinces of Bengal, that we have good reason to complain of the neglect with which they have hitherto been treated. Most writers on the diseases of warm climates either content themselves with merely mentioning them, or else despatch them in just as unsatisfactory a manner as the authors of general systems of medicine. Even Dr. James Johnson, in his excellent work on Tropical Climates, passes them over altogether; and Annesley's voluminous "*Researches*" and "*Sketches*," contain comparatively little on the subject. Mr. Twining, however, surgeon to the general hospital in Calcutta, has given, in the 3d vol. of the Transactions of the Medical Society of that city, a good treatise on Splenalgia.

The chief forms under which Dr. Voigt observed affections of the spleen to occur were, what he terms, *splenitis chronica*, *splenalgia congestionis*, and *lien ingens*.

Chronic splenitis displays a more or less clearly marked inflammatory diathesis, and is attended with pyrexia, considerable pain, and an uncommon degree of weakness: it chiefly attacks children of European origin, and those of rich natives, as well as persons of a more advanced age who are addicted to a stimulating diet; and appears to be principally seated in the proper membrane of the spleen. The swelling, in the first stage, is never very great, and often scarcely perceptible.

Congestion of the spleen (or splenemphraxis) shews plain marks of an atonic diathesis, and is not at all, or but to a trifling extent, attended with febrile symptoms; it does not occasion much pain, but causes a considerable tumour, which occasionally fills the whole of the epigastric and mesogastric regions; this disease most generally occurs among the natives, and is seated in the spongy parenchyma of the organ.

Enlargement of the spleen often occurs and continues for a considerable period without pain or injury to the constitution; it does not appear to be attended with any obstruction, and is observed mostly amongst elderly individuals of the natives.

1. *Chronic splenitis* generally commences with anorexia, restlessness, sleeplessness, and a peculiar irritability of mind, which is especially remarkable in children. They gradually lose all desire for play, wish to be constantly carried, hang the head, cry frequently, and manifest the greatest indifference to all that passes around them. After these precursory symptoms have continued for some days, and sometimes even two or three weeks, the essential symptoms of the disease begin gradually to appear. The face becomes pale, lead coloured, or sallow, and the conjunctiva of a pale bluish colour. The skin, especially of the abdomen, communicates a disagreeable sensation of heat and dryness, and the patient falls into a state of weakness greatly disproportionate to the duration and degree of the attack. The pulse is frequent and somewhat hard, and headach, a peculiar uneasiness, slight difficulty of respiration, palpitations, and occasionally pain in the left shoulder, supervene. There is a constant feeling of tenderness and weight in the left hypochondrium, which is increased by pressure, and that often so much as to cause a loud scream. On examining the patient while lying on his back, and pressing with the fingers under the false ribs of the left side, a hard tumour is felt, which is greater or less according to circumstances, but is always of much smaller dimensions than in cases of splenalgia congestionis. The erect posture is uncomfortable to him; he likes best to lie on his left side, with the body somewhat curved. Although the tongue is moist and clean, he complains of thirst, and, after his meals, of gastrodynia, flatulence, and *ardor ventriculi.* The bowels are very irregular, but generally costive; the fæces are brown, green, or sometimes black; and the urine is pale and copious. After an uncertain period, the disease terminates either by recovery, or by passing into other diseases and causing death.

2. *Splenalgia congestionis* is the most usual of the chronic spleen diseases prevalent about Serampore. Its most remarkable precursory symptoms are lassitude, heaviness, a dislike to any kind of exertion, weakness, costiveness, and a sense of heat in the left hypochondrium. These are followed after some time by a tumour in the region of the spleen, which, though tender to the touch, is by no means so much so as in chronic splenitis. On the other hand, its bulk increases to such a degree that it generally occupies the entire of the left hypochondrium, and of the epigastrium strictly so called, and sometimes of the epigastric and mesogastric regions. The skin is dry and cold, and the cutaneous veins of the abdominal region are considerably dilated and present a reticulated appearance. The patient experiences an indescribable lassitude and weakness, together with vertigo; the lips and gums lose their colour; and the face becomes dingy, sallow, and cachectic. The bowels are tardy and constipated; the fæces dark coloured; the urine pale and copious; the pulse small and feeble; the tongue generally clean and moist, and the appetite good and sometimes even voracious; but the process of assimilation goes on very imperfectly. In proportion as the strength diminishes, the flatulence, anxiety, and irritability increase; the extremities are reduced to skin and bone; cough and dyspnœa set in, and the feet become œdematous. The pulse is sometimes quickened towards evening, and the patient then suffers from an insupportable inward heat, which prevents sleep. He cannot bear to lie upon his right side, and sometimes not even on his back, but finds lying on his left side with the body curved the least uneasy posture. When walking, he generally bends to the left, and supports the left hypochondrium with his hand. The disease in females is generally attended with amenorrhœa. These symptoms, which, with a few exceptions, appear in every case of simple congestion of the spleen, continue till the disease is removed, or terminates in others, and eventually in death.

3. *Enlargement of the spleen* is generally observed amongst elderly natives, and is undoubtedly in most cases a sequela of mild intermittent fevers. The tumour is soft, not of very great magnitude, bears pressure, and produces no inconvenience except a sensation of weight. It does not appear probable that there can be any obstruction existing in such cases, which probably are of the same nature as those mentioned by Regia (Specim. observat. anat. and pathol. Ticin. 1784), in which, according to that author, the vessels can be beautifully injected.

The cachexia connected with *splenalgia Bengalensis* frequently manifests itself by a malignant ulceration, the disposition to which is so great that leechbites and blisters occasionally give rise to foul or phagedenic ulcers, which, in cases where mercury has been employed, and the patient is in a swampy situation and debarred from free access of air, occasionally terminate fatally. *Stomacace* also, both simple and scorbutic, often occurs among the poorer natives; and, although most authors recommend mercury in splenalgia, it is an indisputable fact that even a very small quantity (a few grains for instance) generally occasions a profuse salivation, and occasionally so violent a *stomacace mercurialis*, that mortification sets in, the teeth drop out, the bones become carious, and death ensues.

We have given the description of the symptoms thus at length, as well because it is the most circumstantial that we have met with, as that it may have the effect of inducing the profession in those countries to pay more attention to diseases of the spleen than they have hitherto done. From contemplating a disease in its most exquisite form, as existing where circumstances are most favorable to its development, we are the better able to judge of its pathology, and of its bearings in various respects; and it is not unlikely that, even in this country, many symptoms, generally attributed to dyspepsia, &c. may arise from a milder form of the chronic splenitis above described. We learn too that this disease may exist to a considerable extent without producing any very perceptible tumour; and on the whole it is not unreasonable to conclude that a chronic morbid action, sufficient to produce considerable uneasiness and constitutional disturbance, may be going on in the spleen, for some time, without giving rise to any symptoms of such a nature as to lead us to suspect its seat to be in the organ.

As the author, on account of the prejudices of both natives and Europeans, has not had the opportunities that English medical men attached to the army or to hospitals in India have enjoyed, he is obliged to refer to them for a description of the appearances observed in the spleen after death. Proceeding next to the etiology of the disease, he contradicts Annesley's assertion respecting its comparative infrequency in India, and states that, at least in Bengal, the case is quite the reverse, both according to his own experience and that of Mr. Twining. The predisposing causes he mentions are: 1, season; splenalgia, though occurring at all times of the year, being most prevalent towards the conclusion of the periodic rain, and at the commencement of the cold season: 2, age and sex; children and delicate young men being most subject to it, while elderly persons, and women after puberty, are comparatively exempt: 3, low swampy situations, overgrown with jungle: 4, perhaps sol-lunar influence.

The most usual occasional causes are: 1, tedious remittent and intermittent fevers: 2, want of exercise and of proper clothing, unwholesome diet, and intemperance in eating; these causes often produce an idiopathic splenalgia among the natives: 3, abuse of stimulants, especially arrack: 4, opium-eating: 5, everything that has a tendency to debilitate the system in general, and the digestive system in particular; such, for instance, as depressing passions, &c.: 6, external injuries, such as contusions from falls; but this cause does not often come into play.

In speaking of the proximate cause of splenalgia, there is a remarkable coincidence in opinion between the author and Dr. Hodgkin, in his Essay on the Uses of the Spleen. After stating that it has been proved by the experiments of Defermon on animals, that the spleen is capable of admitting a considerable variation in its size, and that its vessels can bear to be greatly dilated for some time without producing any local or general obstruction, he proceeds to observe that it is evident that, under particular circumstances, in the cold stage of intermittent fever for instance, there is a large quantity of blood thrown in upon the abdominal viscera, which would have a most destructive effect upon the more important organs, if the spleen, from its spongy and elastic texture, were not adapted to serve as a temporary reservoir for a considerable portion of the superabundant fluid: and that repeated and prolonged congestions of this description must, in persons who have been exposed to miasmata and other debilitating causes, produce a state of irritation of the organ which may terminate in chronic inflammation, or in an incurable induration.

*Treatment.* The disease is always difficult to cure, mostly very obstinate, and frequently fatal. Accordingly Dr. V. describes, at a length commensurate with the difficulty and importance of the subject, the treatment that has been found most successful in its different forms; and makes some useful practical remarks on the inconveniences attending other modes that have been proposed by different individuals: the use of mercury, for instance, he most strongly reprobates. The measures he recommends, besides attention to the diet, &c., are to attack the inflammatory symptoms, when such are present, by leeches, gentle purgatives, tepid baths, and the application to the region of the spleen of a cloth dipped in nitro-muriatic acid considerably diluted with water, and covered with an emollient cataplasm; both to be frequently changed.

The febrile irritation and local inflammation once removed, the next indication is to endeavour to diminish the bulk of the spleen. This is most likely to be effected by the continued use of purgatives combined with tonics, and by certain topical applications. He considers the preparations of iron in general, and the sulphate more especially, to have some specific influence on the spleen; but advises caution in its application, as an overdose readily produces cardialgia, gastrodynia, vomiting, and diarrhœa; and it is absolutely contra-indicated where there is any febrile irritation present, as well as in hepatic, pleuritic, and dysenteric complications. The other internal remedies are aloes and myrrh, combined with senna, gentian, carminatives, diuretics, and diaphoretics; nitric acid; and tartar emetic in small doses. The topical applications are, friction with the flesh-brush; nitro-muriatic epithems; small blisters; stimulating liniments to the abdomen; and moxas.

The treatment of chronic splenitis or of congestion of the spleen, difficult enough when they are simple, is rendered still more so by their complication with other diseases. For instance, in *splenalgia hepatica,* mercury, though indicated for the liver, cannot be employed, as it increases the spleen disease.

In another part of the paper Dr. Voigt makes the following remarks on the terminations of splenalgia. "In Bengal, it rarely runs on to suppuration, but, when not fatal, it generally terminates in induration, if not cured in time. The febrile symptoms then disappear, and the pain in the left hypochondrium is much diminished, but the tumour remains and becomes hard and distinct. The health improves, and, with the exception of costiveness, a sensation of fulness and weight under the left false ribs, a dry cough, some dyspnœa, and occasionally a slight pain shooting to the scapula, the patient feels pretty well, and may live on for many years in that condition. He is however generally predisposed by it to fever, liver complaints, dysentery, dropsy, and cholera, by some one or other of which he is at last carried off. Splenalgia, especially when complicated, sometimes runs its course very rapidly, and may terminate in death in three weeks or a month. Œdema of the feet and legs, ascites, dysentery, ecchymoses, a malignant stomacace, and singultus, are the general precursors of death in subacute cases, whilst obstinate diarrhœa and hectic close the scene in the most usual form, splenalgia congestionis. Persons who have once had chronic disease of the spleen are very liable to be attacked by it again.—*Bibliothek for Læger, No.* 2, 1334. Copenhagen.

---

### *On the Use of the Ballota Lanata (Linn:) in the cure of Rheumatic, Arthritic, and Gouty Affections.* By Professor V. L. Brera.

After some questionable remarks on the pathology of rheumatic and gouty diseases, which the author considers to arise from "a supersaturation of the blood and fluids with a morbific matter," and which is relieved by the deposition of its superfluous portion in various parts of the system, under the form of stone, gravel, and other calculous concretions, the author comes to the conclusion that, by the removal of this matter as fast as it is generated, the indications of cure will be fulfilled. He therefore recommends the use of such remedies as free the system from its load by increasing the secretions from the skin, kidneys, &c., and he considers that the ballota lanata is "the most efficacious plant to supply the blood with what is required to relieve it from this morbid supersaturation, and to prevent its reproduction." This plant was found by Pallas and Gmelin in frequent use in Siberia for the cure of dropsy. Rehman, a Prussian physician, introduced it to the notice of Brera, and supplied him with the genuine plant, which he has found of the greatest service in removing by the kidneys dropsical effusions dependent upon visceral congestions.

Finding that many of these cases were connected with gout and rheumatism, he tried it in those diseases also: in which its effects surpassed his expectations; and several other physicians have met with similar success from its use. The results of a number of cases are mentioned; but the following is the only one the details of which are given.

The patient was a sapper and miner, who, from exposure to cold while heated, became affected with most excruciating pain in the pectoralis and mastoid muscles of the right side, and radiating from thence to the dorsal and lumbar muscles. By the use of saline purgatives and diaphoretics, fomentations, prussic acid washes, and oil of hyoscyamus liniments, with tepid baths, the pains were alleviated, without being entirely removed. In the summer of 1831, after two years' illness, the disease increased in violence, and the pains extended to all the joints and to the muscles of the thighs, so that he was unable to raise his arms, close his hand, or move his legs or feet. On September 3d, he began to take the ballota in decoction, made by boiling half an ounce in a pound of water down to eight ounces; the whole to be taken daily. The night following he could not sleep for a burning heat over his body, which about daybreak terminated in a profuse perspiration accompanied by some delirium. With the appearance of the perspiration the pains quite ceased, and he remained at ease the whole of the 4th. He repeated the medicine at night, and the sweating and delirium were more violent than before, but lasted a shorter time. The remedy was repeated the third day, and that night the (critical?) symptoms were much abated. He next experienced a constant and earnest desire to make water, of which he passed a great quantity, fetid, and of a deep reddish orange, and depositing an abundance of sand of the same colour. The pains diminished so much that on the fifth day he got up, and went out of doors on the eighth. The same day the dose of the ballota was increased two drachms, and he continued to take the eight ounces daily, in two doses. The amendment now proceeded more rapidly, so that, on the 20th September, the seventeenth day of treatment, he had recovered the full powers of his body, with an appetite which he had not felt for above a year.

The ballota lanata is indigenous in that part of Siberia which adjoins the Chinese empire. Gathered while flowering, it is brought by the Russians in bales covered with furs. The whole plant is used in medicine. Its smell resembles that of tea; the taste is sharp and bitterish. The cold infusion is not quite clear, and has a greenish yellow colour. The plant imparts all its active properties to weak spirit.

Decoction is the best form of this medicine. It is made by boiling for a quarter of an hour in an unglazed vessel half an ounce of the plant, with as much water as when strained shall amount to eight ounces; this is to be taken at four doses in the twenty-four hours: of course the strength of the decoction must be regulated according to circumstances. It is essential that it should be the Siberian ballota, the effects of that cultivated in gardens being far inferior.

The diuretic powers of the ballota lanata are confirmed by the results of three cases related in the same journal by Dr. Luzzate. The first was anasarca after rheumatism; the next was anasarca after four bleedings for acute bronchitis, in a patient seventy-three years old; the third was rheumatism after delivery, with (milk?) fever.—*Antologia Medica*. No. II. Febbrajo, 1835.

---

### *Alum in Typhoid Fevers.*

PROFESSOR FOUQUIER, one of the physicians of La Charité, is in the habit of prescribing alum, with considerable success, in certain cases of typhoid fever. When the inflammatory symptoms which generally mark the commencement of fever are succeeded by the symptoms peculiar to typhus, such as weakness of the pulse, fixed and dull expression, diarrhœa, acrid heat of the skin, &c., alum is advantageous. If inflammatory symptoms should re-appear, its use is again counteracted; so it is if the bowels (which rarely happens in the second stage,) are constipated; but with these exceptions it may be confidently given, although the most serious nervous symptoms are present. In the stage of collapse, when there is excessive prostration of strength, colliquative diarrhœa, sordes covering the mouth, and fœtid excretions, alum, either alone or with other remedies, acts very beneficially. The diarrhœa diminishes, the tongue becomes moist, and the strength improves. The dose is twenty-four grains daily for three or four days, then increased to half a drachm, and after the same interval to a drachm: when its good effects have been produced, the dose is to be diminished in the same proportion. Gum-water is a suitable vehicle: it may be given in pills, but a solution is preferable.

*Bulletin géneral de Thérapeutique*, Novembre, 1835.

---

### *On the Treatment of Croup by Sulphate of Copper.* By K. G. ZIMMERMAN, M.D.

THE sulphate of copper was first recommended in croup by Hoffmann, (1821;) who prescribed it in the dose of a quarter to half a grain every two hours, and during sixteen years did not lose, according to his own statement, a single patient. Serlo treated from forty to fifty patients with sulphate of copper, and four only died: after venesection, he gave three or four grains as an emetic; following this with a quarter of a grain every two hours. Such was also the treatment adopted by Dr. Zimmerman, except that he generally applied leeches, and only bled from the arm when inflammatory symptoms ran high. He gave the sulphate of copper to fifteen children labouring under well-marked symptoms of croup; and, although the disease in some was very intense, in others far advanced, only two in the fifteen were lost.

CASES. 1. A boy, aged three years, was seized with symptoms of croup, March 9th, 1830. He had had a cough and hoarseness for several days; after exposure to a north-east wind, the symptoms became more severe, and on the evening of the 9th respiration became "crowing." Twelve leeches were applied to the neck, and a quarter of a grain of the sulphate of copper, with sugar, was ordered every two hours. On the 10th, twelve more leeches were applied in the morning. The danger increased, so that in the evening the dose of copper was augmented to half a grain, and twelve more leeches were applied.—11th. One grain of sulphate of copper was given every two hours.—12th. The child died at nine o'clock in the evening, on the fourth day of the disease.

2. A boy, six years old, seized on the 19th of March, took a quarter of a grain of sulphate of copper every two hours, till, after repeated vomitings, all the symptoms of croup disappeared on the 20th. On the 23d, the fifth day of the disease, the boy was perfectly recovered.

3. A stout boy, one year old, was attacked by croup on the 4th of April: leeches were applied to the neck; a quarter of a grain of Cupri Sulph. was given every two hours, and, although this was followed by vomiting every time, eight doses were administered, when the respiration was relieved. The hoarseness gave way on the 13th, the tenth day of the disease.

4. A blond, scrofulous boy, aged three years, had an attack of croup on April 13th: there was a sudden invasion of laryngitis, and the cough was crowing rather than barking. Six leeches over the trachea, and a quarter of a grain of Cupri Sulph. every two hours, were ordered: each dose excited vomiting, but the disease did not yield till the 15th, the fourth day. The hoarseness continued till the eighth day of the disease, when the recovery was perfect.

5. A strong boy, four years old, seen at the commencement of the disease, recovered by taking the sulphate of copper, without previous bleeding. The medicine was, as usual, followed by vomiting.

6. A lively boy, aged six years, seized in the night of the 27th of April, 1833, was not seen till the evening of the 28th, when the symptoms were very intense,—those of laryngitis and tracheitis combined. Venesection to three *tasses* (cups), twelve leeches, and Sulph. Cupr. five grains, were prescribed immediately. At eleven o'clock, sixteen more leeches and a blister were applied; Cupr. Sulph., a quarter of a grain, was administered every two hours: the first powder was followed with relief. On the 30th, the third day of the disease, all the symptoms, except the hoarseness, were gone, which only remained till the ninth day.

7. The same boy had another attack of laryngitis on the 12th of January, 1834; he recovered, under the same treatment, on the 16th.

8. April 27th he had a slighter attack, cured by Cupr. Sulph. alone.—30th. He was quite well, and has not had another attack.

9. A weakly boy, aged six years, had been unwell a week, probably with measles; had a decided attack of croup on the 11th of October, 1833. On the 13th, twelve leeches were applied to the neck; four grains of the sulphate of copper were prescribed, and given afterwards in half-grain doses: in the evening, twelve more leeches and an emetic were prescribed. The Cupr. Sulph. was omitted on the 14th, and five of sulphur were prescribed, &c.; the copper was resumed in the evening. He died on the evening of the 15th, the sixth day of the disease. The trachea and larynx were examined: the mucous membrane was pale, soft, and a few of the vessels were injected; the bronchi appeared filled with a purulent mucus. A younger brother had measles five days afterwards.

10. On the 20th of April, 1834, (a year afterwards,) this brother, now four years old, had an attack of laryngitis. Leeches and the sulphate of copper were employed, and in a few days he was restored.

11. A red-haired strong boy, four years old, seized with croup at 10 P.M. on the 28th September, took the sulphate of copper, and had leeches applied twice: he recovered on the third day of the disease.

12. A delicate blond girl, four years old, had a sudden seizure of laryngitis, from which she recovered in twenty-four hours, after taking four grains of sulphate of copper.

12. A child, one and a half year old, recovered on the second day, after six leeches, Cupr. Sulph. four grains as an emetic, and a quarter of a grain every two hours, had been employed.

Dr. Zimmerman concludes that the sulphate of copper is a very valuable remedy in croup, particularly when conjoined with leeches and blisters. Where there are bronchitis and tracheitis, calomel is preferable; but, in simple laryngitis, the sulphate of copper is advantageous in the majority of cases.

*Hufeland and Osann's Journal*, p. 38—81, B. 2. 1835.

---

### *On Turpentine in Sciatica.* By M. DUCROS, Jun.

M. Ducros has frequently found sciatica, which had resisted the ordinary means, yield to enemata, containing a large dose of the essential oil of turpentine. He has related several successful cases. In one instance, the pain yielded to one enema, containing an ounce of the oil of turpentine: in another, six lavements were administered in three days, when the neuralgia gave way; in a third, the quantity was raised to two and a half ounces in each lavement; in a fourth, the lavements were continued for a fortnight. [We know nothing of the practice ourselves.]

*La Lancette Française*, No. 110, 15 Sept. 1835.

---

### *On Purulent Metastasis.* By Dr. R. FRORIEP.

Dr. Froriep is of opinion that the materials going through the capillaries to the blood are in a state of solution, and do not contain globules; for the porosity of the membrane is not sufficient to permit the passage of globules of pus: so that the appearance of such globules must be coexistent with the effusion of matter. Hence it is incorrect to suppose that a direct discharge of globules of pus takes place into the sac of the pleura, the peritoneum, or the cavity of the arachnoid, as is said, in purulent metastasis; and it is equally erroneous to imagine that, when pus ceases to be secreted from the surface of a sore, it has been transported in the current of the circulation, and deposited in some internal organ, where inflammation is going on.

The mechanism of the formation of the so-named purulent metastasis is as follows: if, during the presence of external suppuration, by any contingency, inflammation comes to be kindled up in some internal organ or other, in consequence of the pre-existing affection it is prevented from displaying the ordinary characteristic train of symptoms; but, attaining imperceptibly its full development, ultimately gains the ascendancy of the previously existing suppurative process, in which the discharge is suspended, while the vicarious inflammation, acting upon an organism already enfeebled by disease, pursues a rapid course towards a purulent termination.

In support of this mode of explanation, Dr. F. refers to what has been frequently observed, under such circumstances, on post-mortem examination; particularly to those cases where no deposition of pus is discoverable,—merely a serous exudation, or simply the phenomena of incipient inflammation without the effused products.

In accordance with this view, it is plain that a tonic and stimulant treatment, for the purpose of compelling the pus to flow internally, as is vulgarly said, can only serve to augment the mischief commencing. The measures pursued must be antiphlogistic, proportioned to the strength and constitution of the patient.

*Wochenschrift f. d. Heilkunde,* No. 8. *Berlin,* 1834.

---

### *On the proper Temperature of Sinapisms.*

The volatile oil, on which the stimulating properties of the powdered seeds of mustard depend, is not disengaged or formed unless water is added to them; but it has been imagined that very hot water was preferable to cold. M. G. Fauré, sen. has proved, by many careful experiments, that this is not the case, but that water, when heated to 190° (F.) and upwards, prevents the disengagement of the volatile principle of mustard: consequently, that sinapisms should be made with cold water, and for foot-baths the powdered mustard should be first mixed with some cold water, to which boiling water should be added, to raise it to the necessary temperature. By one of those coincidences which are not uncommon, the same facts have been simultaneously discovered by MM. Geiger and Hesse in Germany. The most satisfactory rationale is, that the sudden heat coagulates the vegetable albumen which forms a coating to each molecule, that prevents the water acting upon it. All causes which coagulate albumen produce the same effect, such as alcohol, strong acids, &c. Cold water, on the contrary, dissolves the vegetable albumen. *Journal de Pharmacie, Septembre,* 1835.

---

### *On Salicine as a Substitute for Quinine.*

[The following cases were treated at the medical *clinique* of Prague during the years 1832 and 1833, and are reported by Dr. A. M. Pleischl, Professor of Chemistry. As they are authentic and satisfactory illustrations of the therapeutic agency of a remedy but little known in this country, we give an abridged translation of them.]

Case I. The patient, a weekly girl of fourteen, was attacked with ague on the 12th of May, 1832. As there were symptoms of congestion of the liver and spleen, leeches, &c. were employed; and, on the 26th, the salicine was given in the dose of two grains every two hours; but as this did not suffice, after the third attack the dose was increased to four grains every two hours; after which there was no paroxysm. As the liver and spleen were still tumid, the salicine was continued, together with a decoction of tamarinds containing cream of tartar; poultices were also applied to the parts, and the patient was discharged cured on the 16th of July.

Case II. A strong female servant, aged twenty-two, and of a sanguine temperament, was attacked by convulsions during the sixth month of pregnancy, and then by a tertian ague. She was treated with the sulphate of quinine in very large doses, but without permanent advantage. The quantity given at last was five grains every hour, with one-sixth of a grain of ipecacuanha. An intermittent hematemesis now set in; and, as this, though mitigated, was not cured by opium, it was thought right to omit the quinine, and give salicine instead. The dose was five grains every three hours; and, although there were still three fits of hematemesis, the last two were hardly worth mentioning, and the patient was cured of her obstinate intermittent. For fear of a relapse, the salicine was continued for several days: the patient took in the whole about six drachms.

Case III. A youth of eighteen was cured of a tertian ague, under which he had laboured for three weeks. After an interval of a fortnight, he had another attack, which his physician quelled by an emetic. Ten days had scarcely passed away, when he was attacked for the third time. The paroxysms were irregular, and, as opium was given in vain *in scopum prævertentem,* four grains of salicine were ordered to be taken every four hours during the day of intermission. The next attack was anticipatory, being an hour earlier than its time: there were no premonitory symptoms; the cold stage was much shorter and milder, lasting barely half an hour; the hot stage lasted four hours, and was succeeded by a profuse sweat of three hours' duration. The salicine was continued. The second paroxysm consisted of a rigor, followed by gentle heat of scarcely fifteen minutes' duration, and a sweating stage of three hours. This was the last fit, and there was no relapse.

Case IV. A robust servant girl, aged twenty-two, had suffered for several months from a violent cough, which tormented her especially before daybreak. She then had a gastric tertian fever of an anticipatory type; and, having committed an error in diet during her convalescence, had a relapse. The gastric symptoms being now quieted, but the fever remaining, she was ordered four grains of salicine three times a day, and took three of these powders on the day of intermission likewise. The next paroxysm was slight, and was the last. She took in the whole fifty-six grains of salicine, and had no relapse afterwards.

Case V. An active servant girl, aged twenty-four, with a proclivity to congestions and inflammations, after an error in diet was attacked with a tertian ague complicated with considerable gastric symptoms. On each day of fever there were two paroxysms; the first took place on the 9th of May, and on the 20th the patient entered the medical *clinique,* having had six days of fever, and twelve paroxysms.

While measures were taken against the gastric symptoms, the fever made its attack on the morning of the 21st, the evening paroxysm was slight and transient. The patient was now ordered to take three grains of salicine three times a day: the ague did not return, but the salicine was continued for a few days to guard against a relapse, and with the desired effect.

Case VI. A servant, aged thirty-nine, who was labouring under acute ascites, was attacked during its course by a headach, which returned at the same hour every day. It was a pressing and tearing pain in the frontal and temporal regions. The patient complained besides of heat in the head, palpitation, and exhaustion. The skin was very hot, and the pulse somewhat quickened, but no other symptom of fever was present. Four grains of salicine given in the evening, and the same quantity the next morning, made the subsequent attack very trifling indeed; but still the remedy was repeated once more. The patient remained free from headach for five days, and then had another paroxysm, which was subdued by salicine, as before. Five doses, of four grains each, proved sufficient to master this intermittent headach, and prevent any relapse.

Case VII. A servant girl, aged twenty-eight, who had twice suffered from ague, which had left a tumid liver, was attacked with very violent headach during the course of an *Impetigo erysipelatodes.* It returned every day at the same time, and lasted for six hours. The salicine, which had shown its efficacy in this form of disease, was given in five-grain doses in the evening and in the morning before the paroxysm. There was one more paroxysm, and afterwards a mere shadow of one, and the two powders having been again administered, the headach returned no more.

Case VIII. The patient was an athletic coachman, aged thirty-six, and was afflicted with intermittent face-ache. The pain followed the ramifications of the ophthalmic portion of the fifth pair of nerves. He had suffered from this frightful disease twice before during the last six years, and this, the third attack, was owing to taking cold after recovering from the influenza. At seven o'clock every morning, the following precursory symptoms made their appearance: formication, following the course of the above-mentioned nerve on the right side; anxiety; general increase of warmth; exhaustion, and throbbing headach. There soon came on the most violent cutting, tearing, and pricking pains, particularly in the course of the frontal nerve, and the pain gradually extended itself over the whole right half of the face. The patient tossed about in his agonies, and had a horror of being touched, especially above the eyebrows, and even avoided speaking, as it increased his pain. He complained of transient stabbing pains in his eye, and it seemed to him as if the eye was pushed out of the orbit; the intolerance of light was so great, that he closed his eyelids almost convulsively, and the pain was unbearable if they were opened by force; in addition, the secretion of tears was increased, and the paroxysm was accompanied by perfect blindness, which gradually went off afterwards. The whole face was distorted and flushed, with an increase of *turgor;* the pulse, too, was somewhat quickened, and the skin hotter than usual, but no other disturbance of any system. The symptoms continued to decline till towards noon, and in the afternoon the patient was quite well. Leeches had been applied to the right temple, and an anti-rheumatic regimen adopted at the patient's dwelling, but without success. The patient being now in the practical school, the salicine was administered; four grains being given in the evening, and four more in the morning before the attack. After two paroxysms the doses were doubled. The next paroxysm was mitigated both in severity and duration; yet, with the hope of preventing the following paroxysm entirely, three eight-grain doses were now exhibited, one at eight in the evening, one at six in the morning, and one just before the usual hour of the attack. Some slight pain still remained, and therefore the quantity of salicine was increased to half a drachm divided into three doses, which were taken within twenty-four hours. A more violent paroxysm followed, upon which two scruples were given, which prevented any farther attack. The patient left the hospital perfectly cured.

These cases are remarkably satisfactory, and would seem to show (the second especially) that salicine is far from being a mere substitute for quinine. Dr. Pleischl enters into some details concerning the prices of salicine and the sulphate of quinine, and observes that the latter is more than twice as costly as the former. He concludes by asserting, that, even if salicine were much the dearer of the two, it would be better to use it, because it is of home manufacture, and can be got in war as well as in peace. *Medicinische Jahrbücher des K.K. ö. Staates,* 1835.

---

### *Case of Poisoning by Arsenic, successfully treated by the hydrated Tritoxide of Iron.* By M. Geoffroy.

F., æt. 36, hairdresser, during an attack of delirium tremens, took from his desk a paper which contained arsenic, threw the poison into a glass, poured water on it, stirred it up with his finger, and drank it. Finding that some of the poison remained in the glass, he added more water, and was about to drink it, when one of two persons who were in the room, wishing to see what he was drinking, looked at the paper from which he had taken the powder, and seeing that it was marked arsenic, endeavoured to prevent his drinking it, but did not succeed; and he not only swallowed the liquid, but put into his mouth, with his finger, the powder which remained in the glass. M. Geoffroy arrived immediately afterwards, and, on discovering that arsenic was contained in the paper, ordered several glasses of sugared water to be given immediately, and procured, as soon as possible, some hydrated tritoxide of iron. In twenty minutes this was obtained, and four or five pints of warm or cold water, charged with it, were given in a quarter of an hour. This produced copious vomiting and a large stool. For the next seven or eight hours this treatment was continued, and the patient vomited and was purged three times. There was neither colic, heat in the throat, nor any symptom of poisoning; he complained of cramps in the fingers, and during the whole time was delirious, talking and gesticulating. The quantity of drink was then diminished: he slept during the night, and in the morning appeared well.

It was ascertained that he had swallowed a drachm and a half at least of white arsenic. From twenty to twenty-five pints of water had been given, in which was suspended the oxide of six ounces five drachms of the sulphate of the tritoxide of iron.

[This is a valuable case, as there was no doubt of the enormous dose of poison taken.] *Journal de Médecine et de Chirurgie pratiques. Sept.* 1835.

---

### *Hydrated Peroxide of Iron as an Antidote to Arsenic.*

MM. Bineau and Majesté, of Saumur, relate the following cases, to prove the efficacy of the hydrated peroxide of iron as a counter-poison to arsenic in the human subject. On the 13th of August last, about two o'clock, five little girls, on leaving school, ate part of a cake, containing one-fifth of its weight of white arsenic, which had been prepared to kill rats.

L. D., æt. 7, who had eaten a piece weighing about two drachms, had, half an hour afterwards, pain in the throat, a sensation of strangulation and vomiting, succeeded by pains in the belly, great thirst, faintness, incessant restlessness, and spasms. Dr. Bineau saw her at four o'clock: she had then vomited five times, and rejected sugared water and milk which she had swallowed. He gave her one grain of tartar emetic, which produced vomiting and two stools. In an hour he had prepared the tritoxide of iron, and by nine o'clock he had given five ounces, in divided doses. During this period she vomited five times, and had one black fætid stool: there were stupor and slight convulsions of the limbs. At ten o'clock these symptoms were relieved; she slept quietly, and was well the next day.

M. G., æt. 5½, swallowed about three drachms of the cake, and a quarter of an hour afterwards vomited. Between this time and five o'clock she vomited twenty times, and rejected all fluids. After vomiting, faintness and depression, followed by extreme restlessness, pain over the whole body (particularly in the belly and legs), cold perspirations, livid face, great thirst. At four o'clock there was great and constant stupor, without loss of intelligence. At five o'clock, M. Bineau administered five or six drachms of the hydrated tritoxide of iron, and repeated the dose frequently at first, and gradually increasing the intervals until ten o'clock. During this time she vomited only three times, and the pain ceased; but there was constant and alarming stupor and depression. At ten o'clock the pulse rose, and at four she slept naturally. In the morning she had two stools. No subsequent symptom.

The other three cases were treated by M. Majesté.

Marie B., æt. 7, and Louise, her sister, æt. 5, each ate about two drachms of the cake. At four o'clock M. Majesté saw them: the face of the eldest was contracted, pale or livid, eyelids injected, great thirst, very hot skin, pulse 120, belly tympanitic and painful, particularly the epigastrium, general depression, and constant vomiting and purging, since the poison had been taken. The symptoms of the younger were rather milder. M. Majesté returned to his dispensary, and in less than an hour prepared twelve ounces of the hydrated tritoxide of iron, and he gave to each two ounces at four doses, in twenty minutes. The vomiting ceased, but returned in an hour, when he gave two ounces more at longer intervals, and a lavement with half an ounce. At eight o'clock the vomiting returned with colic, and an ounce was given to each, with half an ounce in a lavement: the vomiting ceased, and did not return. They passed a good night. With the exception of some little intestinal irritation, and an eruption in the eldest, which yielded to simple treatment, there were no other symptoms.

The other child, aged 9, was less violently affected. The vomiting ceased on taking the antidote, and in eight hours after the attack all danger was over.

*Remarks.* The remedy was prepared in the following manner:—One ounce of iron filings, with four ounces of nitric acid and four ounces of muriatic acid, were introduced into a large glass vessel, and subjected to a gentle heat until the iron was dissolved; to this solution sixteen ounces of cold distilled water were added, and after some minutes the metal was precipitated by introducing two or three ounces of liquid ammonia. The vessel was then filled with common water, agitated, and the whole filtered. This left about twelve ounces of the hydrated tritoxide of iron. A teaspoonful of this weighed about an ounce. The process occupied about an hour, and it was immediately repeated. Its action as an antidote was evident. The dose in each case was very large, above thirty grains of arsenic; and, although vomiting took place, yet a much smaller quantity has been often known to kill, although there was vomiting. The symptoms were always immediately relieved by the iron. The antidote itself appears harmless, as from four to six ounces were given to each child without any ill effects; and, as this is the case, it is advisable, even long after the poison has been taken, to estimate the quantity rather by its effect on the symptoms, than by any proportion to the poison. It is advantageous that this antidote is tasteless.

*Journal des Connaissances Médico-Chirurgicales, Novembre,* 1835.

### *Fumigations in Hooping-Cough.*

Dr. Dohm, of Heide, in the duchy of Holstein, has accidentally discovered a remedy for hooping-cough, that promises to be of considerable use in that too-often obstinate and dangerous disease. Two of his own children, a boy and a girl, (the former one, and the latter three, years old,) had been suffering from hooping-cough for between two and three months; during which time several remedies, including belladonna, had been tried in vain. The paroxysms were very frequent and extremely violent, so that the fæces and urine used sometimes to be expelled involuntarily. An accident of this kind occurred one evening during the absence of the father; and, to remove the ill smell thereby occasioned, the bedroom was fumigated, and that to such an extent that the child was enveloped in the smoke. Contrary to the expectation of the doctor, the child had not another attack that night; the cough became much milder, and the repetition of the same treatment soon cured it. This encouraged him to try it in other cases, and he invariably found the paroxysm greatly relieved by it, if not completely stopped. The fumigation was made with the common *species fumales* of the Pharmacop. Slesvico-Holst. (Olibani libr. duas, Benzoes, Styr. Calamitæ, sing. libr. dimid., Flor. Lavendul., Rosar. rub., singul. unc. quatuor.) He [we think, very justly,] considers the benzoin to be the most efficient ingredient.

*Pfaff's Mittheilungen, 1ste Jahgr., 1 und 2 Heft.*

